# Assessing the acceptability of a text messaging service and smartphone app to support patient adherence to medications prescribed for high blood pressure: a pilot study

**DOI:** 10.1186/s40814-020-00666-2

**Published:** 2020-09-21

**Authors:** Aikaterini Kassavou, Charlotte Emily A’Court, Jagmohan Chauhan, James David Brimocombe, Debi Bhattacharya, Felix Naughton, Wendy Hardeman, Cecilia Mascolo, Stephen Sutton

**Affiliations:** 1 Department of Public Health and Primary Care, The Primary Care Unit, Behavioural Science Group, University of Cambridge, Cambridge, UK; 2 Tees, Esk and Wear Valley Hertfordshire Partnership NHS Foundation, St Albans, UK; 3 Department of Computer Science and Technology, Mobile Systems Group, University of Cambridge, Cambridge, UK; 4 School of Pharmacy, University of East Anglia, Norwich, UK; 5 School of Health Science, University of East Anglia, Norwich, UK

**Keywords:** Hypertension, Primary care, Digital intervention, Acceptability, Medication adherence

## Abstract

**Aims and objectives:**

This paper describes a pilot non-randomised controlled study of a highly tailored 56-day text messaging and smartphone app prototype intervention to increase adherence to anti-hypertensive medication in primary care. The aim of this study was to evaluate the acceptability of the intervention and obtain patients’ views about the intervention content, the delivery mode, and the mechanisms by which the intervention supported medication adherence.

**Methods:**

Patients diagnosed with hypertension were invited and recruited to the study via general practice text messages and attended a face to face meeting with a member of the researcher team. Participants were asked to test the text messaging intervention for 28 consecutive days and switch to the smartphone app for 28 more days. Participants completed baseline and follow-up questionnaires and took part in semi-structured telephone interviews. Digital log files captured patients’ engagement with the intervention. Participant transcripts were analysed using thematic analysis. Descriptive statistics were used to summarise data from questionnaires and log files. A mixed methods analysis generated data to respond to the research questions.

**Results:**

Seventy-nine patients expressed interest to participate in this study, of whom 23 (64% male, 82% above 60 years old) were registered to take part. With one drop-out, 22 participants tested the text messaging delivery mode (with 20 being interviewed) and four of them (17%) switched to the app (with 3 being interviewed). All participants engaged and interacted with the text messages and app notifications, and all participants found the intervention content and delivery mode acceptable. They also self-reported that the interactive elements of the intervention motivated them to take their medications as prescribed.

**Conclusion:**

This study provides evidence that the digital intervention is acceptable by hypertensive patients recruited in primary care. Future research could usefully investigate its feasibility and effectiveness using rigorous research methods.

**Trial registration:**

ISRCTN12805654

## Key messages

- The acceptability of a digital intervention, combining text messaging service with a smartphone app, to support medication adherence in patients with hypertension in the UK primary care, has not been evaluated.
- This pilot study proved that the digital intervention is acceptable among patients with hypertension, and it recommended that an interactive intervention to enable patients track health-related behaviours might be the way forward to provide personalised and highly tailored advice and support for adherence in primary care.
- Future trial should investigate the feasibility of the digital intervention to support medication adherence and associative blood pressure in patients with hypertension, as an adjunct to usual care consultations.

## Introduction

In England, over 12.5 million people are diagnosed with hypertension (high blood pressure) [1]. High blood pressure is a major risk factor for morbidity and mortality [2]. Taking anti-hypertensive medication as prescribed can significantly reduce these risks [3]; however, a substantial proportion of patients do not take their medication as prescribed. A recent meta-analysis showed that 41% of people do not adhere adequately to anti-hypertensive medications, i.e. they take less than 80% of their prescribed tablets [4]. Non-adherence contributes to increased hospital admissions, additional consultations, referrals, investigations, and medicine wastage. Improved adherence could save of just over £100 million per year; thus, the Department of Health recommends that novel interventions for medication adherence should be developed and tested [5].

Non-adherence to prescribed medication may occur for a number of reasons, like patients forgetting or missing a dose or a day of their medication. These reasons are described as non-intentional non-adherence (NINA) or intentional non-adherence (INA) [6]. Interventions are likely to be more effective to support medication adherence, when they address either or both of these determinants by providing highly tailored advice to the individual [7]. Tailored interventions include behaviour change techniques (BCTs) mapped onto these key determinants. BCTs are assumed to be the ‘active ingredients’ of behavioural interventions [8]. For example, INA can be addressed by reinforcing positive beliefs about taking medications (e.g. ‘keep control of your blood pressure today by taking all your tablets as prescribed’) and by countering negative beliefs and concerns (e.g. ‘please do not forget to take your tablets, even when you do not have any symptoms. Pills have been prescribed to lower your blood pressure’). NINA can be addressed through explicit and implicit reminders [9]. Both NINA and INA can be addressed by simple query messages to report behaviour (e.g. ‘have you taken your medications as prescribed during the past 7 days?’).

Digital interventions like text messaging and downloadable applications on smartphones (apps) are promising ways to provide advice, reminders, and encouragement, and to support patients to take their tablets as prescribed. Our recent meta-analyses suggested that automated telephone-based interventions, including text messaging interventions [7] and apps [10], double the odds for adherence to medication prescribed for long-term health conditions. Our recent feasibility trial found that digital interventions, including text messaging, can provide highly tailored advice to address either or both NINA and INA for medication adherence and is feasible and potentially effective adjunct to primary care consultations [11]. However, there is no intervention that has combined text messaging with a smartphone app, both to maximise the reach of the intervention and to utilise the advantages of the automated tracking technology embedded into smartphone apps when facilitating a highly tailored behaviour change intervention.

Based on our previous promising findings [11], we have developed a 56-day prototype intervention, which we call PAM (Programme on Adherence to Medication), delivered by a text messaging service followed by a smartphone app. This pilot study aimed to pre-test its acceptability to support medication adherence in patients with hypertension in primary care. To our knowledge, no other text messaging followed by a smartphone app intervention has been pre-tested to address non-adherence to medication in the UK primary care setting.

Qualitative and quantitative data was integrated into a mixed methods analysis to answer the primary research question of this pilot study: is an individually tailored text messaging service followed by a smartphone app acceptable to support medication adherence by patients treated for hypertension in primary care? Our secondary research questions were to explore the acceptability of the intervention content and delivery mode functionalities, and the mechanism by which these might have influenced medication-taking behaviour.

## Methods

### Recruitment methods and procedure

Three primary care practices were recruited in this study, which were located in East of England and at diverse deprivation areas. Patients were deemed eligible to participate in this study if they (a) had a diagnosis of hypertension or high blood pressure, (b) were prescribed at least one anti-hypertensive medication for a duration of at least 3 months before recruitment, (c) be had poorly controlled hypertension (e.g. readings of blood pressure > 140/90 mmHg recorded in practice databases during a period of 6 months before recruitment), (d) were aged 18 years or older, (e) had a good understanding of English, (f) owned and regularly used a mobile phone, and (g) had the capacity to provide informed consent. We aimed to recruit 25 participants to respond to our research questions.

Across the three recruited practices, a total of 1340 patients eligible to take part in this study were identified from practice records. A member of the practice staff sent one text message invitation to all eligible patients. The invitation included a link to access the study material, and to register interest to participate, online. In total, 79 patients responded to the text message invitation with an interest to participate during the first week from the day invitations were sent, 54 of whom in the first 2 days. From those, the first 23 patients who met all the eligibility criteria to participate were enrolled in the intervention during a baseline meeting with a member of the research team. During the baseline meeting, a member of the research team obtained written informed consent and facilitated the collection of baseline data. Two researchers conducted the recruitment meetings from January until March 2019.

### Intervention

Participants were provided with the 56-day prototype intervention, which involved text messaging support for 28 days and then switching to the smartphone app for the consecutive 28 days. All patients were asked whether they would like to switch from the text messaging to the app intervention, after they have completed the text messaging intervention. The app intervention was compatible with android phones only. 

Participants received daily reminder messages with explicit advice (e.g. ‘please do not forget to take your medication today: amlodipine, 2 tablets, 5mg’), daily advice messages (e.g. ‘the health benefits of taking your meds regularly is having low blood pressure. Please keep looking after yourself by taking your medications’), and query messages (e.g. ‘have you taken all your prescribed meds in the last 7 days/ today? Reply Yes or NO’). The app included additional functions; provided participants with an option to request and receive feedback on behaviour and to change the delivery of the intervention messages. The app tracked medication related routines without feedback to the patient.

### Data collection methods and procedure

Data were collected using questionnaires, telephone interviews, and digital log files.

#### Questionnaires

The questionnaire included questions regarding patients’ intention to adherence and beliefs about taking medication. Intentional (INA) and non-intentional non-adherence (NINA) was measured using the 5-item MARS questionnaire [12], and beliefs about adherence were measures using 10 items measuring necessity beliefs (e.g. ‘If I were to take all my medications as prescribed without missing a day it would do more harm than good’), control beliefs (e.g. ‘taking my medications as prescribed keeps my blood pressure under control’), affective attitudes (e.g. ‘taking my medications as prescribed every day without missing a day is pleasant/unpleasant/neither’), social norms (e.g. ‘If they were prescribed tablets, most people whose opinion I value, would take all their prescribed tablets without missing a day’), copping self-efficacy (e.g. ‘I take all my medications as prescribed without missing a day, even if I am busy at home’), and generic emotional state (e.g. ‘How much of the time during the past month have you felt calm and peaceful’). The tailoring questionnaires were adapted from, and developed based on our previous studies [11, 13]. Patients’ answers to these questions were used to tailor the content of the advice messages.

The questionnaire at baseline collected information about patients’ prescribed medications (e.g. name of medications and dosage), and about the delivery of the intervention i.e. preferred time to receive the intervention messages. Patients’ answers to these questions were used to tailor the content of the reminder messages.

Patients completed questionnaires at baseline and at follow up. Baseline questionnaire collected information about patients’ demographic characteristics. Follow up questionnaires obtained views about the acceptability of the intervention. Baseline questionnaires were completed by patients during the meetings with a member of the research team and follow-up questionnaires were complete using an online webpage, which was emailed to patients.

#### Interviews

Semi-structured interviews were chosen to obtain in-depth accounts of participant experiences, thoughts, and beliefs. Detailed answers were provoked by the researcher, who used open-ended questions and prompts to explore participants’ experiences with using the intervention [14].

During the interviews, a member of the research team used a semi-structured interview guide to prompt patients’ views about the intervention content and delivery modes, and obtain recommendations for improvement (please see Appendix 1 and Appendix 2 for the semi-structured interview guide). Each participant was provided with the option of a weekly 15-min telephone interviews or one follow up 45-min telephone interview. Seventeen patients opted for the weekly calls, and the other five for the interview at the end of the trial. Interviews were audio recorded, and the audio files were transcribed by a third-party company. Patients who could not attend the interviews (*n* = 1) provided their feedback using emails. Interviews were conducted from February until April 2019.

Weekly data collection was selected to capture participant thoughts and opinions within its present context as opposed to potentially weeks later, thus deriving more detailed, richer data from each participant. Regular data collections also aimed to identify and solve any technical issues that may arise when piloting an innovative digital intervention.

#### Digital log files

The engagement with the intervention was captured by digital log files. Digital log files captured the intervention content participants received and interacted with (e.g. reported whether or not the medication was taken, check feedback on adherence to medication, used app settings to tailor message delivery). Additionally, digital log files captured medication related routines. The log files are documents detailing each participants’ recorded actions and responses whilst using the intervention and were utilised as objective measure of intervention usage and engagement. Data from log files were extracted by a member of the research team.

### Analysis

Two members of the research team used thematic analysis and analysed all interview transcripts independently. The coding for emerging themes was deductive and aimed to provide answers to our research questions. Inductive themes were considered as recommendations for improvement. The two researchers compared notes and codes for each interview transcript and made a mind map to visually link quotes and codes to one another. The two researchers discussed in-depth themes that had emerged from the data. Nvivo software was used to facilitate data analysis.

Data from log files were coded by a member of the research team and integrated into the analysis. Data obtained from questionnaires and digital log files were summarised using descriptive statistics. A mixed method approach was used to synthesise quantitative and qualitative data to provide answers to our research questions [15].

## Results

**Fig. 1:**
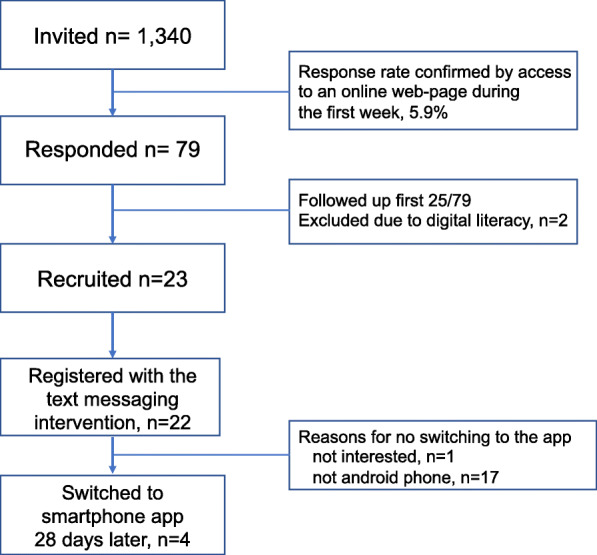
Study flow chart

Seventy-nine patients answered to the text message invitation and expressed their interest to participate in the study (5.9% response rate to invitation), of whom the first 23 patients attended baseline meetings and provided written informed consent to participate. All 23 initially recruited patients completed the baseline questionnaire (100% completion rate), which included the tailoring questions and measured medication adherence. One patient dropped out of the study before registering with the intervention due to personal reasons. In total, 22 participants enrolled in the intervention: all registered with the intervention for 28 days using the text messaging service, four of them (17%) selected to switch to the app, and two installed the app and used the intervention for 56 consecutive days (see Fig. 1).

Twenty patients (out of the final 22 participants) completed the follow-up interviews (90.9% response rate), and 19 completed the follow-up questionnaires (86% completion rate). Participants were 64% men and 82% above 60 years old (see Table 1).

**Table 1:** Practice (*n* = 3) and participants’ (*n* = 22) characteristics

Practice area Index of Multiple Deprivation rank^a^, patients with hypertension/doctor ratio^b^, patients with hypertension /staff ratio (excluding doctors)^c^	Participants			
	Patient ID	Age bracket	Gender	Participated at follow-up^d^
7, 11350/5, 11350/10	1	70–79	Male	Yes
	2	60–69	Female	No
	3	30–39	Female	Yes
	4	60–69	Male	Yes
	5	50–59	Female	Yes
	6	50–59	Female	Yes
	7	70–79	Male	Yes
	8	70–79	Male	Yes
	9	60–69	Male	Yes
	10	70–79	Male	Yes
	11	70–79	Male	Yes
12, 13445/6, 13445/8	12	40–49	Female	No
	13	60–69	Male	Yes
	14	60–69	Female	Yes
	15	60–69	Female	Yes
	16	60–69	Male	Yes
	17	70–79	Male	Yes
	18	60–69	Male	Yes
12, 3553/7, 3553/13	19	60–69	Male	Yes
	20	70–79	Female	Yes
	21	70–79	Male	No
	22	60–69	Male	Yes

^a^The Index of Multiple Deprivation in England, ranks from 1 (most deprived area) to 100 (least deprived area)

^b^Includes GPs

^c^Includes nurses, health practitioners, and healthcare assistants

^d^If a participant provided complete data at follow up. 'Yes' inidicates completion, 'No' indicated non completion

The data analysis generated results in three main themes to provide answers to our research questions:

1. Acceptability and usage of the components (i.e. content and delivery functionalities) of the prototype digital intervention to patients prescribed medications to treat high blood pressure
2. Mechanisms by which these components might have supported medication adherence
3. Recommendations to improve the acceptability of the intervention

### Acceptability and usage of the digital intervention in patients with hypertension

All participants interviewed (*n* = 20) reported that the text messaging service was easy to use (see Table 2 quote 1 and Fig. 3). Similarly, patients who used the app reported the app functionalities were easy to navigate (see Table 2 quote 2 and Fig. 3).

**Table 2:** Thematic analysis results

**Theme 1. Acceptability of the digital intervention for patients diagnosed with high blood pressure**		
**Quote**		**Participant ID, age, and tested delivery mode**
1	“No, no, everything was straightforward really. There was nothing at all that stood out as being awkward about it, or difficult.”	Participant 7, M, age 70–79, text
2	“Logical, smooth interface, easy to navigate.”	Participant 9, M, age 60–69, text and app
3	“No, it’s just a straightforward message, have I taken all my meds? Which I suppose if I hadn’t, it would have made me think, well have I?”	Participant 1, M, age 70–79, text
4	“P: It’s almost patronising” “I: Okay, so do you have any suggestions of how we might try and change that so it’s not so as annoying or patronising? P: Well maybe less frequency”	Participant 16, M, age 60–69, text
5	“Because it’s a different that was every day, you know it’s not going to be the same message so you’re going to pay attention and read it, and reading it should reinforce in your mind ah, I need to take my tablets.”	Participant 9, M, age 60–69, text and app
6	“This tab (with feedback on behaviour), for me personally, did I stick to the right times, how far off was I, how was I on, do you know what I mean? That sort of information, for me, would have been yes, great. Say after about a week or a month you could look and think what’s going on here?”	Participant 18, M, age 60 to 69, text and app
7	“I: Would you have any reservations about us using GPS data? P: None at all. I think that’s probably better than messages coming through when you’re out and when you’re driving or shopping, say you say. It’s rather intrusive. If anything comes through when I’m driving, it has to wait until I’ve finished driving. I don’t even acknowledge it.”	Participant 1, M, age 70–79, text
8	“Simply because the greater the likelihood of getting the message, I don’t know, would equivocally improve delivery of the message.”	Participant 14, F, age 60–69, text
**Theme 2. Mechanisms by which the digital intervention has supported medication adherence**		
**Quote**		**Participant ID, age, and tested delivery mode**
9	“I: So, it makes you think back onto the past week and reflect? P: Yes, yes. And you think, let me look at my tablets, to see if there’s as many left as there ought to be?”	Participant 1, M, age 70–79, text
10	“Whereas, if it goes off in your pocket and you think I will get it after, but about 5 o’clock or something it goes off in your pocket and you think oh, I better get this. Have you taken your medicine? No, I haven’t, so then I’ve got to then go upstairs, view it and then press yes. So, then it’s forced on me, if you like, to make sure I do take it.”	Participant 18, M, age 60 to 69, text and app
11	“I was waiting for the alarm to go off. Then it was about 20 minutes late, but I was already there with the tablets to take anyway. So, it’s made me take them, even though I didn’t get the alarm at eight o’clock, if you see what I mean. So, that was fine. So, I had taken them anyway because that’s made me more aware that I should take them at that time rather than any time, at random times.”	Participant 15, F, age 60–69, text
12	“P: Well, yes just exactly what I mean, being committed to take your tablets as prescribed because it’s for your own benefit.”	Participant 1, M, age 70–79, text
13	“I do think it does help if people feel as though they’re involved (with taking their medications)”	Participant 20, F, age 70–79, text
14	“To me, that’s saying have you taken it yes or not, I suppose you could lie about it but if you’re going to do that what’s the point of having the text message, I don’t see the point. But yes, it’s an interactive thing isn’t it? So, you’re taking ownership, if you like and dealing with it that way.”	Participant 18, M, age 60 to 69, text and app
15	“P: What’s the next one? ‘Tablets are part of your self-care.’ I think that was the last message. It’s a reminder that the ultimate responsibility lies with yourself, so take ownership… People don’t take enough personal responsibility. So, if you like, that was quite a good reminder – well actually your meds are down to you and nobody else. You are prescribed them, but you’ve got to take them.”	Participant 9, M, age 60–69, text and app
16	“If you just get a reminder all the time and it goes off in your pocket, you think it’s just a text, I’ll answer it later. If you think that oh, that could be about my medication and I’m going to have to respond yes or no, then you take it out and you’re going to look at it and you’re taking ownership of your responsibility obviously it’s your anyway for taking your meds.”	Participant 18, M, age 60 to 69, text and app
17	“I: Okay, is there anything else that you wanted to speak about regarding the messages at all, like the query message style message? P: No, no. Like I say, I think, to have that, sort of, as a summary of how has the week gone? Have you taken your tablet? Yes. Well done. And, like I say, in a strange sort of way, it’s very motivational. I: Well that’s good. So, did it motivate you, and how did it motivate you? P: Well I think you sort of smile when you get the ‘Well done’ and (inaudible 00:10:19) the following week. And I actually did manage to take my tablet every day, on time. So, it just sort of … it’s like an added encouragement.”	Participant 13, M, age 60–69, text
18	“But yes, it’s an interactive thing isn’t it? So, you’re taking ownership, if you like and dealing with it that way.”	Participant 18, M, age 60 to 69, text and app
19	“I think the advice part is like kind of key, but also I think, even though it's text message and it limits you to characters, but I think giving succinct points, people are more likely to read them, so maybe on the advice have one topic a week and every day, come up with a different aspect of that topic”	Participant 5, F, age 50–59, text
**Theme 3. Recommendations to improve the acceptability of the digital intervention**		
**Quote**		**Participant ID, age, and tested delivery mode**
20	P: Well, the negative sides makes you think oh god, you know, you start to get a little bit thinking that oh, …. if you see what I mean, but not, you know, but if it’s then positive, you think yes, that’s why I need to take them.	Participant 15, F, age 60–69, text
21	“I: If you were designing the service, was there anything that you would do differently? P: Well, the only thing that I could suggest would be, although it might be a bit long-winded, if you’ve got morning medication and evening medication that you would perhaps need, like I’ve said before, be more likely to forget the evening one than the morning one because it’s not in my routine so much. But I didn’t get a message for the evening one, so could possibly need a message for both situations. I: Okay. And would you want those messages to come through separately at the times you take your medication, or would you want one sort of big text message in the morning reminding you of all the medications that you need to take that day? P: Yes, it would have to be at different times, I suppose, because if I had a message about the morning one and the evening one in the morning, I’m still likely to forget if I was going to in the evening one. So, you’d need two messages a day and I suppose if you’re going to be taking lots of things at different times that might become a little troublesome, I suppose.”	Participant 22, M, age 60–69, text
22	“And they have am and pm on them, or lunch time one as well. So, you could do it three times a day because most people it is only three times a day for medication. Morning, lunch time, and evening, isn’t it?”	Participant 6, F, age 50–59, text
23	“I: So, if the reminder came through at a time where you couldn’t take your medication, did you just press no or did you use the snooze button to snooze? P: To be honest, on the app? I: Yes, on the app? P: I didn’t know there was one. I never saw that.”	Participant 18, M, age 60 to 69, text and app
24	“I think help-wise, like a how to maybe, because it’s obviously complicated, a how to guide maybe, that could probably help.”	Participant 18, M, age 60 to 69, text and app
25	“I didn’t really understand, like looking at this (feedback on behaviour) tab and bits and pieces like that …”	Participant 18, M, age 60 to 69, text and app
26	“I: Do you wish that we maybe used a bar chart, maybe something more visual or was it okay for you? P: No. well, everybody is different, aren’t they? Some people prefer, some people learn by visualise, look at it, other people like the colours, like the graphs, other people prefer data as it’s written down.”	Participant 18, M, age 60 to 69, text and app
27	“P: Probably because, I mean I much easier to just do it on a daily basis becomes sometimes you can be forgetful, unless you write it down, I don’t know. Maybe it might make people anxious about their memory and stuff like that, I don’t know. As much as it has a clinical purpose, why do you need to ask them at the end of the week when it’s just better to ask them every day since their short-term memory is probably much more reliable? I: Yes, that’s true. So, you would suggest asking, having that query message sent every day instead of once at the end of the week? P: Yes.”	Participant 14, F, age 60–69, text
28	“P: I’d probably put a bit less advice in, it might get a bit boring after a while, it’s okay the first week they were all different, I’m sure that if that goes on for months and months and months you’d have to repeat some of them quite regularly. I: So, less frequent advice messages? P: All I really would need is a reminder. I: So, the reminder every day and how often would you have the advice message come through? P: Probably once a week would be good.”	Participant 11, M, age 70–79, text
29	“The advice. Not the one reminding you to take your medication, the other one after that. Maybe once or twice a week would be alright, to put something like that out. But getting that every day, seemed to be a little bit too much really…. No. I just think, at my stage, to keep getting advice messages like that, it would lose the point of it, it would lose its impact then ... I just think if it was less frequent, then maybe it would have more of an impact.”	Participant 7, M, age 70–79, text
30	“What I don’t have any problem with is somebody who comes up front and says this app is going to do and it’s going to watch where you go because, because, because. So, yes, I have concerns, but as you’ve explained that, I have no concerns at all because you’ve been upfront about it.”	Participant 9, M, age 60–69, text and app
31	“I: Okay, so is it that NHS name or that label of the NHS that makes you think (it's secure)? P: I think it would be the label. I: So, the labelling of the NHS makes you feel a lot safer with the data? P: Yes. I: Okay, similar too, would you feel the same if it was the University of Cambridge logo instead of the NHS? P: I don’t see why not. I don’t see it being a problem, because I mean you’re with them now and we’re discussing it aren’t we, so I don’t think that would be a problem. It might be for some people.”	Participant 15, F, age 60–69, text
32	“I personally think, because I’m 70 next year, but in my head, I’m only 50, if you know what I mean. But a lot of people who are my age are forgetful. So, if you’re leading a busy life, I would say at least six weeks, and I’m in week three, but because I get up early in the morning and everything, I’m busy doing things, I tend to forget if I’m sitting in front of the television or something. So, yes, I think six weeks would be a good routine because as I said, I’ve done it in three where I’ve been, oh god, I must take my tablets, it’s eight o’clock. So, I personally think perhaps six weeks would make them more aware that they should be doing this, and that would built a routine.”	Participant 15, F, age 60–69, text
33	“P: So, you don’t feel as if actually they do take care with what’s happening. It sounds very negative, I don’t mean it quite like that, but I think it’s just a question that they probably would feel worthwhile and that someone understands that they are taking tablets you see. Because I never see the same doctor. I: So, do you think that this service could maybe help to counteract some of those feelings of being…? P: Oh yes, definitely.”	Participant 15, F, age 60–69, text

#### Reminder messages

**Fig. 2:**
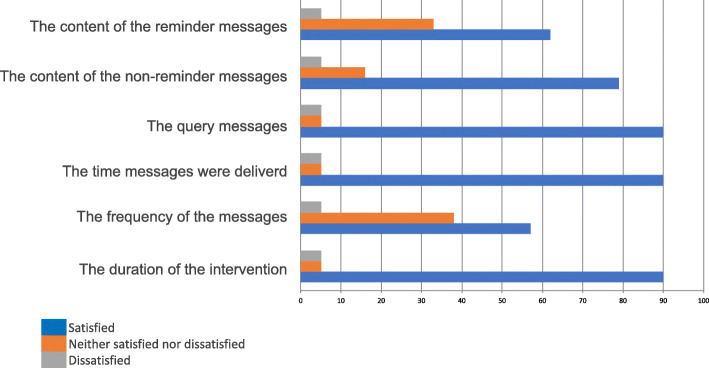
Satisfaction with the intervention. *N* = 19 patients. Data collected by follow-up questionnaires

**Fig. 3:**
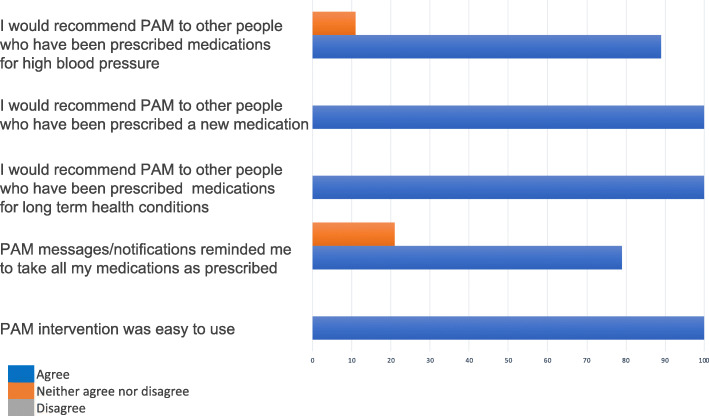
Acceptability of the intervention as an adjunct to usual care. *N* = 19 patients. Data collected by follow-up questionnaires

Commenting on the reminder messages, one participant thought the simple message sufficed and also allowed for the participant to ‘double-check’ that they had taken their medication that day (see Table 2 quote 3). Most participants reported the reminder messages as acceptable and a positive aspect of the digital intervention (see Figs. 2 and 3). However, few participants reported disliking the frequency of the daily reminder messages, with one participant finding them overbearing. To overcome this challenge, participants recommended that the intervention should include more options for participants to reduce the frequency of receiving these messages, especially if the intervention was of a longer duration (see Table 2 quote 4). Both app patients have used the snooze functionality of the app to re-schedule the time of the reminder messages (see Table 3).

**Table 3:** Intervention engagement per delivery mode

Text messages	Frequency
Report whether or not the behaviour was performed	72
**App**	
Report whether or not the behaviour was performed	52
Feedback on behaviour	
Daily	15
Weekly	9
Tailor messages delivery	
Snooze reminder messages	5
Update refill reminder day and time	7

Engagement with the intervention for the duration of 28 days. *N* = 22 patients for the text messaging. *N* = 2 for the smartphone app, after the text messaging intervention. Data extracted by digital log files

#### Advice messages

Participant opinions on the advice, non-reminder, messages were much more variable (see Fig. 2). For example, one participant suggested that the advice messages increased his attention to read the messages and in turn increased his motivation to take his medication as suggested (see Table 2 quote 5). This may be due to receiving a variety of advice messages throughout the digital intervention, with each message emphasising and addressing a different reason regarding medication adherence. Data from digital log files suggested that participants who used the app acknowledged or dismissed the receipt of all advice messages.

#### Query messages

Furthermore, the ‘query’ message was sent to participants asking them to reply ‘yes’ or ‘no’ as to whether they had taken all their prescribed medication. Participants were asked to respond to the query message one per week (when testing the text messages) or when each medication was taken (when switching to the smartphone app). The concept of the query message arose from the behaviour change strategy of ‘reporting whether or not the behaviour was performed’ [7, 11, 16]. All participants reported to query messages and most reported that they liked these messages (see Table 3 and Fig. 2). All participants received the query messages and the response rate, to both text message and app notifications, was 100%, and this engagement score was maintained throughout the intervention, indicating high participant engagement with the digital intervention and acceptability of these messages to support medication adherence.

Participant discussed the benefits of the ‘feedback on behaviour’ functionality on the app, which generates a percentage score of patients’ self-reported medication adherence over the past day and week (e.g. ‘you’ve reported that you have taken 89% of your prescribed medication last week’). Participants found this intervention content informative and reported using it to self-monitor their medication adherence and to reflect on their patterns regarding their medication-taking routine (see Table 2 quote code 6). Both app users requested the feedback on their self-reported behaviour by checking the app functionality, in most cases after they have self-reported medication adherent behaviour (see Table 3).

#### Functions to tailor message delivery

This function provides the participant with options to re-schedule the frequency (e.g. by texting 'less' to receive less messages or 'more' to receive more intervention messages) and the time of the reminder messages (e.g. by snoozing the reminder notifications). Participants tailored the delivery of the reminder messages on average 5 times when using the smartphone app, and none when using the text messages. This function was found useful to those participants who reported that were busy at the time the reminder message appeared and could not respond confirming whether or not they have taken their tablets. Patients have also the option to set and re-schedule the time and date of their refill prescription reminders, and both patients set their refill reminders and used the app function to re-schedule the time and day of the refill reminder notification on average 7 times, during 4 weeks (see Table 3). In all occasions, they tailored the delivery of the reminder messages successfully, implying the importance of including this function in the app, as well as how easy it is to use it. Some participants suggested that the option to tailor the delivery of the refill reminder notifications increased the acceptability of the intervention.

#### Acceptability of tracking medication related routines using the smartphone technology

All participants were asked about their views about the app using the GPS, Wi-Fi, and accelerometer embedded on their phone, to automatically track their behaviours related to medication taking during the intervention. All participants reported having no concerns over the security of their behavioural patterns related to medication adherence being captured through the app sensing technology. Some of them have also suggested that tracking technology could make daily medication taking effortless and easier (see Table 2 quotes 7 and 8). They also suggested that the information about their medication taking routines, collected by smartphone technology, could be utilised during consultations with health care providers to improve medication adherence.

### Mechanisms by which the intervention has supported medication adherence

For some participants, receiving the query message was an opportunity to reflect on their medication-taking behaviour and raise their awareness as to whether or not they had taken all the doses of the medication they were prescribed (see Table 2, quotes 9, 10, and 11, and Fig. 3). This strategy seemed to have motivated medication taking through several ways: (a) by increasing participants’ commitment to reply to the query message, (b) by raising awareness of tablet-taking routine, (c) by increasing feelings of involvement with their own medication-taking routine, and (d) by empowering them to take their medications as prescribed (see Table 2 quotes 12, 13, and 14). These findings suggested that reporting behaviour is a motivational behaviour change technique, which can lead to increased performance of the target behaviour.

The importance of accessing feedback regarding medication adherence behaviour in motivating participants to change their behaviour, was also suggested by participants testing the app. Both app testers checked this app functionality after successfully installing the app (see Table 3), with one of the participants attempting to view their adherence report six times in the first day of switching to the app. This could be explained by an initial exploration period after first installing the app; however, both participants continued to check the functionality with feedback on behaviour at least once a day for 4 days after switching to the app. These findings imply that participants find this function interesting and an engaging feature of the app and that feedback on the behaviour might be important to increase patients’ motivation to change behaviour.

It was also found that the tailored daily advice messages may have caused an increase in the participants’ attention and curiosity when interacting with the intervention, and therefore an increase in engagement with the intervention. It was also recommended that the tailored advice messages have prompted self-monitoring and possibly increased acting upon these advice messages.

A number of participants agreed that the digital intervention may also help to increase patients’ feeling of empowerment and ownership of their long-term health condition, with one participant expressing how he particularly liked one of the advice messages which encouraged patients to take responsibility for their own medication-taking routines as a self-care process. Participants reported that they found the intervention messages particularly useful because they suported them to establish a sense of ownership over the condition and motivated them to be more self-aware of their independent responsibility over their health condition (see Table 2 quotes 15, 16, 17, and 18).

All participants liked the interactive elements of the intervention, and reported feeling motivated to continue taking tablets regularly and as prescribed, after interacting with the intervention (see Table 2 quote 15).

Most participants expressed preferences towards the advice messages which included positive reinforcement to take their tablets (see Table 2 quotes 17, 18, and 19). The preference towards the advice messages came up multiple times during the interview, specifically in the context of increasing patients’ motivation to take their medication to treat hypertension as a self-care process (see Table 2 quotes 15 and 19).

### Recommendations to improve the acceptability of the digital intervention

All patients reported that they would recommend the intervention to other people who have been prescribed medications for long-term health conditions and those newly prescribed (see Fig. 3). They have also made recommendations to improve intervention content, delivery, and implementation procedures.

Many participants suggested receiving separate reminder messages for every medication they take each day. However, many other participants reported patients who are prescribed multiple daily medications a potential limitation to this idea, for example sending individual reminder messages to an individual who takes ten or more tablets every day will most probably be less acceptable (See Table 2 quote 20 and 21). However, another participant recommended a solution to this problem, suggesting combining the medication reminders into morning, lunchtime, and evening routines (see Table 2 quote 22).

Furthermore, one of the participants testing the app reported not using the additional app functions, which could be used to tailor the delivery of the medication reminder until a later time (see Table 2 quote 23). The participant recommended a help guide with interactive elements to explain all features of the app and how to use them when first installing the app (see Table 2 quote 24 and 25). A help guide to aid patients’ navigation through the app would potentially increase accessibility and intervention engagement.

Participants also recommended different ways to summarise the feedback on behaviour, such as graphs or colour-coded systems and traffic light colour code systems (green = goal met; orange = room for improvement; red = goal unmet, improvement needed), which are universally understood and easy to interpret, thus might be accessible to a range of individuals with varying learning styles (see Table 2 quote 26).

Moreover, the frequency of the query message was also discussed between participants; some participants suggested that responding to the query message every day would be effortless and thought others would prefer less frequent queries about medication adherence. However, another participant suggested receiving a daily query message, stating that it would help those with poor memory or those with busy daily routines (see Table 2 quote 27).

Towards the end of the 28-day intervention, many participants suggested once a week, instead of every day, was an appropriate frequency to receive the advice messages to improve intervention acceptability and engagement (see Table 2, quote 28). However, one participant expanded upon this idea and suggested that the impact of the advice messages might be decreased if received daily (see Table 2 quote 29). Thus, receiving one or two advice messages a week would be more acceptable to encourage patients to take their prescribed medications regularly, especially if the intervention was to support sustained medication adherence.

The tracking functions of the digital intervention could facilitate effortless medication adherence in the long term, however to increase its acceptability, a clear and honest communication is needed to explain the privacy and the purpose of using tracking data to support medication adherence (see Table 2 quote 30). Furthermore, some participants also suggested using the logo of the NHS or University of Cambridge to visually link the app to a trusted institution (see Table 2 quote 31).

Overall, participants suggested that the digital intervention could support medication taking daily routines and be an acceptable adjunct to primary care and increase satisfaction with the health care provided by GP practices (see Table 2 quotes 32 and 33 and Fig. 3).

## Discussion

This mixed methods study suggests that the PAM prototype intervention is acceptable by patients with hypertension and that it may support medication adherence by improving motivation to take all doses of the prescribed medications as part of a daily routine. It was found that the content of the highly tailored intervention was acceptable and that all participants used and engaged with the intervention. The study also supported that all patients interacted with the query intervention messages to support their medication-taking behaviour and they found the additional tracking functionalities of the smartphone technology acceptable, with few (17%) opting to use these additional tracking elements to support medication adherence.

The high response rate to the messages enquiring participant to report about adherence to medication demonstrates a promising projection of engagement that will be especially important for the future trial of this intervention, to provide information about how intervention engagement associates with changes in behavioural and clinical outcomes. Thus, not only do the results of this study suggest that both the text messaging and app delivery modes of the digital intervention are acceptable, but also that the content of the intervention is acceptable by patients with high blood pressure. Therefore, the positive results from this study provide us with confidence to proceed into testing this digital intervention for feasibility at a randomised controlled trial.

A strength of this pilot study is that it utilised two digital delivery modes to maximise the reach of a highly tailored intervention even to those with low digital literacy and to provide with a choice of an automated self-tracking advice to support medication adherence. To our knowledge, this is the first intervention that utilised a combination of text messaging and smartphone app and has been pre-tested to address non-adherence to medication within the UK primary care setting. Another strength of this study was its unique data collection method of weekly interviews that allowed us to identify and solve any technical issues more explicitly and quickly than using the traditional hour-long interview at the end of the trial. This study also used a mixed methods analysis and synthesised qualitative and quantitative data from multiple sources to respond to our research questions.

This study is limited by the small number of patients as well as the short duration of the intervention. Another limitation of the study is the small proportion of patients switching to the smartphone app.

## Conclusion

The findings of this study informed the upcoming trial to evaluate the feasibility of this digital intervention to support medication adherence and blood pressure to patients prescribed treatement for hypertension in primary care. This study provides evidence that this novel digital intervention is acceptable by patients with hypertension in primary care. Not only is the content of the intervention acceptable, but also the two digital delivery modes were found to be acceptable by patients. This study also found the digital intervention to be highly engaging and supportive to patients. When considering the high cost medication non-adherence imposes upon the NHS, this study is of particular importance as it provides evidence supporting that this low-cost intervention may be an acceptable answer to help achieve healthcare priorities.

## Data Availability

All data generated or analysed during this study are included in this published article. Additional information is available from the corresponding author on reasonable request.

## Appendix 1

**Interview schedule for face-to-face interviews with people with high blood pressure to assess their experiences using the text message delivery mode of the digital intervention**

**Interview set-up**

- Begin audio recording device
- Ask participant for their consent to be interviewed about their experiences using the intervention, and their consent for this interview to be audio recorded

## Introduction

- Introduce myself
- Give an overview of the aims of the interview
- Consent issues
- Ask if participants have read the information sheet and if they have any questions
  - If not, overview the information included
- Clarify that they can withdraw from the interview at any time, without giving a reason
- Clarify that the interview will be audio recorded, explain the use of transcripts and the confidentiality issues

**Questions to assess participants’ experiences using the digital intervention**

- Did the digital intervention help you to take your medications as prescribed?
- If yes, in what way?
- If no, can you elaborate on the reasons?
- What did you like most about the digital intervention?
- Do you ever forget to take your medication or alter the dose?
- If yes, which medications?
- If yes, how often would you estimate you do not take your medications as prescribed?
- What might be the reasons for this?
- What did you find difficult about the digital intervention?
- How did you find the content of the reminder messages?
- Did you like the personalisation of the text messages? E.g. name.
- How did you find the content of the advice messages?
- Do you have any other suggestions for what we may include in the advice messages?
- Did you find any faults in the text messaging service?
- Do you have any suggestions for improvements regarding any features in the text messaging service?
- How easy was it to reply to the Q&A style questions on the digital intervention?
- If you were designing the digital intervention, what would you do differently?

**4th Interview**

- All of the above questions, plus:
- We’re also developing an app to deliver this service. Do you think you would use an app version of this service?
- Who do you think would use the app?
- We could include other features in the app such as tablet-taking logs, percentage of medication adherence, and a snooze button for the reminders. Do you think these ideas would be helpful to you when encouraging you to take your tablets?
- What else do you think would be helpful to include into the app?
- The app may include ‘sensing data’, which means it may look at the accelerometer data on your smart phone, and will not send you messages when you are exercising. Do you think this is acceptable? Would you be happy for the app to look at this data?
- Also, the app could look at when your phone is connected to your home Wi-Fi and only send you messages when you’re at home with your tablets. Would you be happy for an app to do this?
- What are your concerns with the sensing data?
- Finally, would you like to test using the app for one month?

### Appendix 2

**Interview schedule for face-to-face interviews with people with high blood pressure to assess their experiences using the app delivery mode of the digital intervention**

**Interview set-up**

- Begin audio recording device
- Ask participant for their consent to be interviewed about their experiences using the intervention, and their consent for this interview to be audio recorded.

## Introduction

- Introduce myself
- Give an overview of the aims of the interview
- Consent issues
- Ask if participants have read the information sheet and if they have any questions
  - If not, overview the information included
- Clarify that they can withdraw from the interview at any time, without giving a reason
- Clarify that the interview will be audio recorded, explain the use of transcripts and the confidentiality issues

**Questions to assess participants’ experiences using the app**

- Did the app help you to take your medications as prescribed?
- If yes, in what way?
- If no, can you elaborate on the reasons?
- What did you like most about the app?
- What did you dislike about the app?
- Do you ever forget to take your medication or alter the dose?
- If yes, which medications?
- If yes, how often would you estimate you do not take your medications as prescribed?
- What might be the reasons for this?
- What did you find difficult about using the app?
- How did you find answering whether you had taken your medication every day?
- Was it acceptable to you to answer every day?
- Should we include reasons why you may have not taken your medication so you can track these reasons?
- Did you use the snooze button at all?
- Did you find it easy to use the snooze button?
- Is the snooze button a helpful addition to the app?
- Did you like the advice messages coming through every day?
- Do you have any other suggestions for what we may include in the advice messages?
- Did you look at your adherence reports?
- Were they easy to understand?
- Did looking at your adherence report change your motivation for medication adherence?
- What else do you think we could include for increase motivation to adhere to medication?
- What other reports would you like to see? Reports of other behaviours? Different style of reports e.g. pie chart, bar graph, line graph.
- Who do you think would benefit from using the app?
- If you were designing the app, what would you do differently?
- What else do you think would be helpful to include into the app
