# Correction to: Assessing the acceptability of a text messaging service and smartphone app to support patient adherence to medications prescribed for high blood pressure: a pilot study

**DOI:** 10.1186/s40814-020-00698-8

**Published:** 2020-10-07

**Authors:** Aikaterini Kassavou, Charlotte Emily A’Court, Jagmohan Chauhan, James David Brimicombe, Debi Bhattacharya, Felix Naughton, Wendy Hardeman, Cecilia Mascolo, Stephen Sutton

**Affiliations:** 1 Department of Public Health and Primary Care, The Primary Care Unit, Behavioural Science Group, University of Cambridge, Cambridge, UK; 2 Tees, Esk and Wear Valley Hertfordshire Partnership NHS Foundation, St Albans, UK; 3 Department of Computer Science and Technology, Mobile Systems Group, University of Cambridge, Cambridge, UK; 4 School of Pharmacy, University of East Anglia, Norwich, UK; 5 School of Health Science, University of East Anglia, Norwich, UK

**Correction to: Pilot Feasibility Stud 6, 134 (2020)**

**https://doi.org/10.1186/s40814-020-00666-2**

Following publication of the original article [1], the authors reported an error in the name of the fourth author. The surname should be Brimicombe.

The original article has been updated.
